# Supplementary material for: Discovery of an Adaptive Neuroimmune Response Driving Itch and Fast Tick Removal with Implications for Preventing Pathogen Transmission
Source: bioRxiv. 2025 Aug 28:2025.08.22.671835. Preprint. [Version 1] doi: 10.1101/2025.08.22.671835 (PMC12443053; doi:10.1101/2025.08.22.671835)
Supplement: 1 [file NIHPP2025.08.22.671835V1-supplement-1.pdf]

bioRxiv preprint doi: https://doi.org/10.1101/2025.08.22.671835; this version posted August 28, 2025. The copyright holder for this preprint (which was not certified by peer review) is the author/funder. This article is a US Government work. It is not subject to copyright under 17 USC 105 and is also made available for use under a CC0 license.

SUPPLEMENTAL INFORMATION

Figure S1

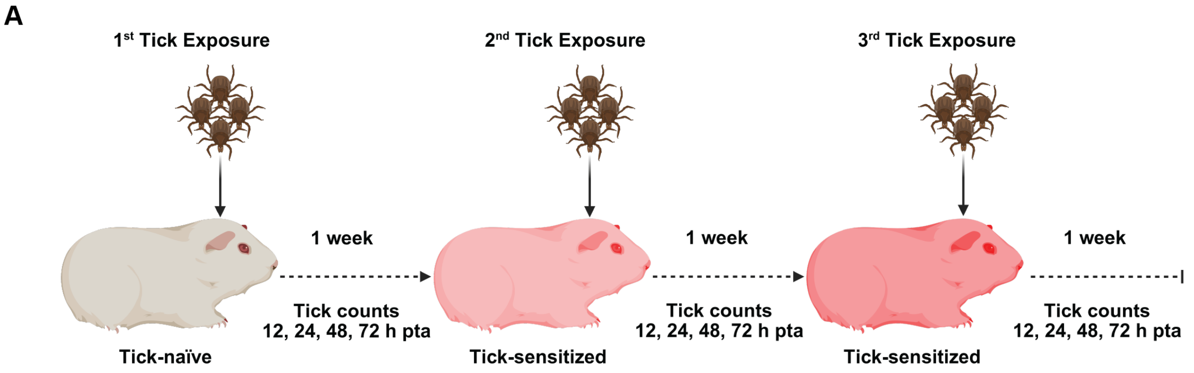

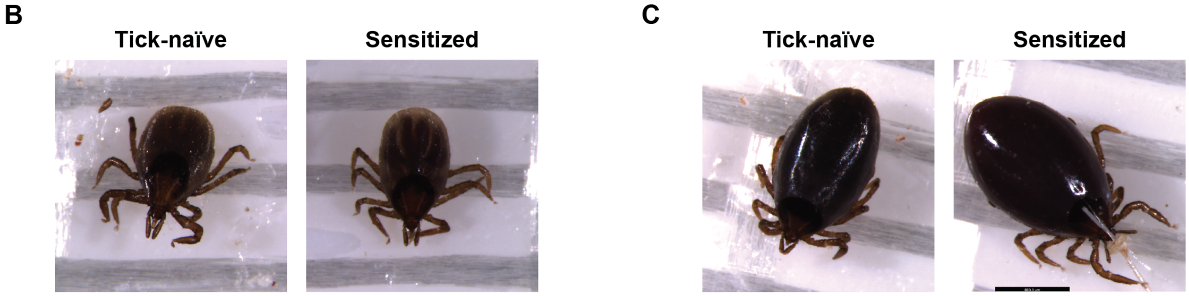

48 h pta	Tick-naïve	Sensitized
Alive	40	37
Dead	11	9

72 h pta	Tick-naïve	Sensitized
Alive	52	45
Dead	4	9

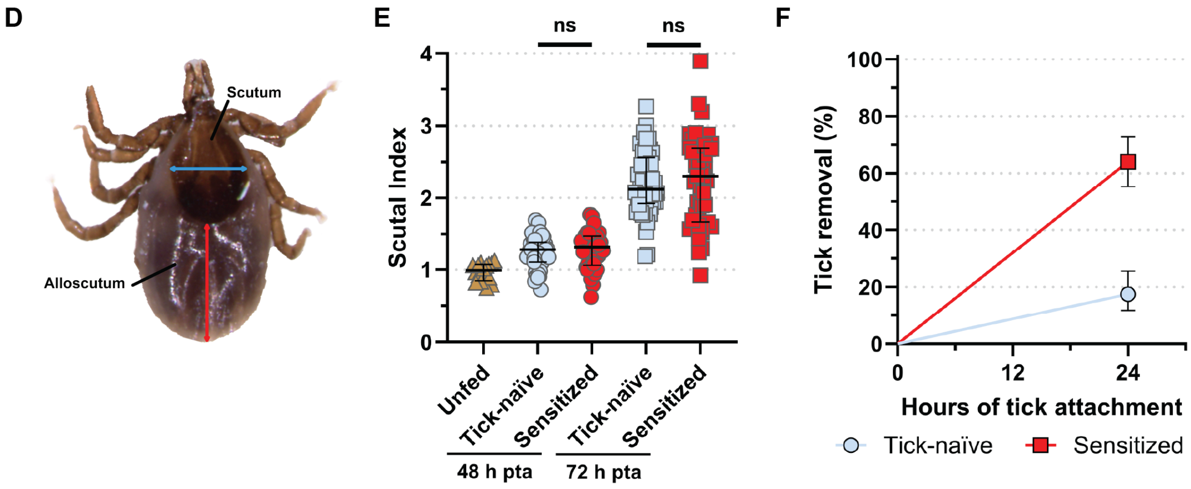

bioRxiv preprint doi: https://doi.org/10.1101/2025.08.22.671835; this version posted August 28, 2025. The copyright holder for this preprint (which was not certified by peer review) is the author/funder. This article is a US Government work. It is not subject to copyright under 17 USC 105 and is also made available for use under a CC0 license.

Figure S1. (**A**) Repeated tick exposure experimental design (Created in BioRender: https://BioRender.com/47zaovj)**.** (**B,C**) Images of ticks fed on sensitized and tick-naïve guinea pigs (GPs) with a respective cross table of live/dead tick counts at 48 and 72 h post tick attachment (pta). Cross table analysis by (**B**) Chi^2 test (p=1), and (**C**) Fisher's Exact test (p=0.5124). (**D**) The scutal index is a ratio produced by dividing the alloscutum's length by the scutum's width. (**E**) Scatter plot: Scutal index values of nymphal ticks fed on tick-naïve (48 h pta: *N*=42, 72 h pta: *N*=50) and sensitized (48 h pta: *N*=38, 72 h pta: *N*=48) GPs, or unfed (*N*=16*)*. Two-way ANOVA (excluding unfed): Sensitization state (tick-naïve / tick sensitized) p=0.3229, Time (48 h pta versus 72 h pta) p<0.0001. Median ± IQR shown. (**F**) Smoothed, inverted Kaplan-Meyer plot showing probability of tick removal (%) during a 1st (Tick-naïve, *N*=120 ticks) and a 4th (Sensitized, *N*=114 ticks) tick-exposure at 24 h pta. Analysis by Peto & Peto modification of the Gehan-Wilcoxon test: p<0.0001. 95% CI shown. For detailed statistics see supplementary report.

bioRxiv preprint doi: https://doi.org/10.1101/2025.08.22.671835; this version posted August 28, 2025. The copyright holder for this preprint (which was not certified by peer review) is the author/funder. This article is a US Government work. It is not subject to copyright under 17 USC 105 and is also made available for use under a CC0 license.

Figure S2

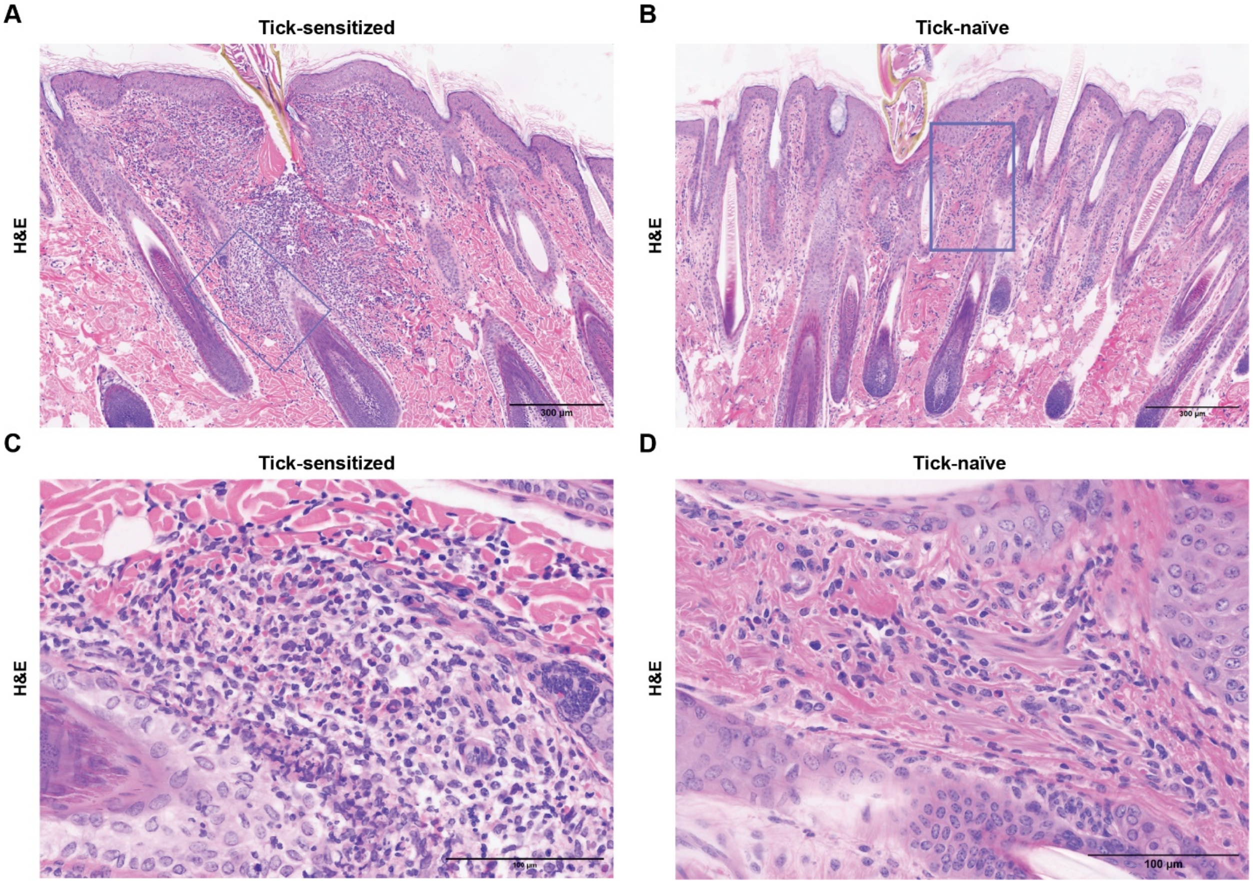

Figure S2. (**A,B**) Representative H&E skin cross-sections (>|5µm|<) of tick bite sites 24 h post tick attachment (pta) in (**A**) three times weekly tick-sensitized and (**B**) first-time exposed tick-naïve guinea pigs (GPs). (**A**) Significant focally extensive dermal inflammation consisting primarily of a mononuclear cell infiltrate surrounding and separating adnexal structures. (**B**) Mild epidermal hyperplasia with a minimal dermal infiltrate. (**A,B**) Scale bar: 300 µm. 10x (**C,D**) Enlarged section from (**A,B**) to illustrated (**A**) presence and (**B**) absence of eosinophils in tick bite sites. Scale bar: 100 µm.

bioRxiv preprint doi: https://doi.org/10.1101/2025.08.22.671835; this version posted August 28, 2025. The copyright holder for this preprint (which was not certified by peer review) is the author/funder. This article is a US Government work. It is not subject to copyright under 17 USC 105 and is also made available for use under a CC0 license.

Figure S3

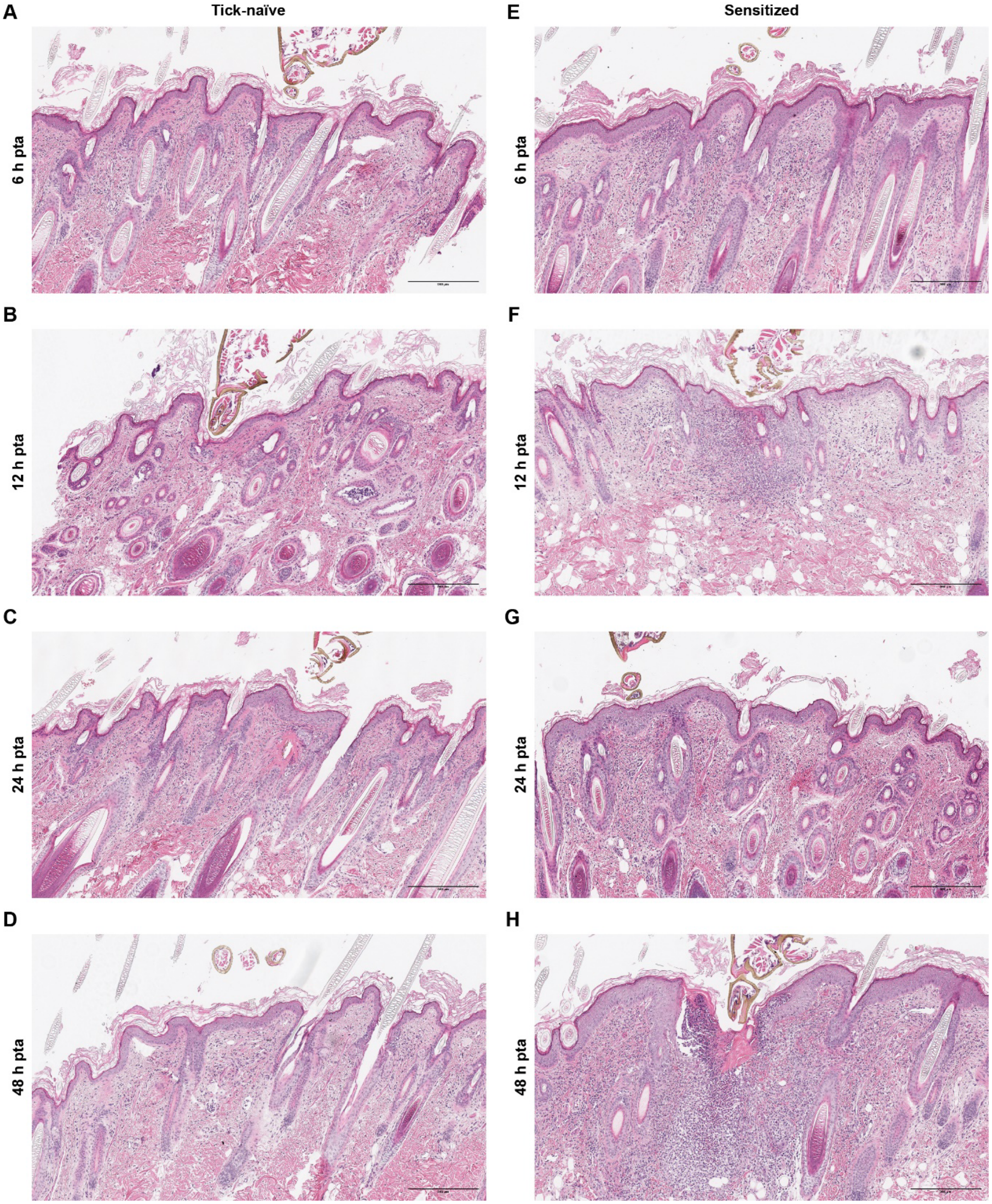

Figure S3. Representative histological sections (>|5µm|<) stained with H&E showing cell infiltrates at the tick bite sites in tick-naïve and tick-sensitized GPs over 48 h pta. Scale bar 300 µm. 10x.

bioRxiv preprint doi: https://doi.org/10.1101/2025.08.22.671835; this version posted August 28, 2025. The copyright holder for this preprint (which was not certified by peer review) is the author/funder. This article is a US Government work. It is not subject to copyright under 17 USC 105 and is also made available for use under a CC0 license.

Figure S4

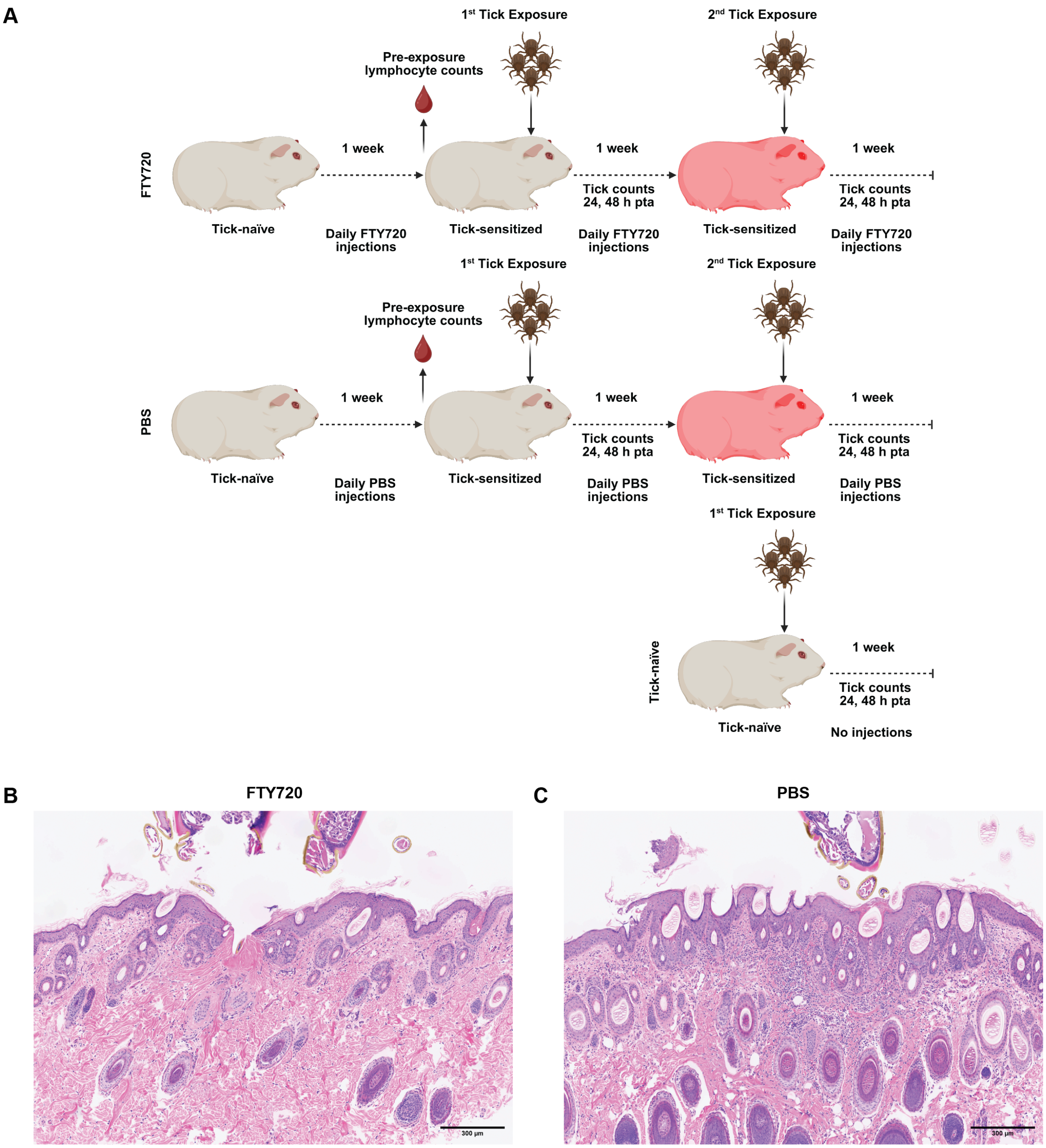

Figure S4. (**A**) FTY720 experimental design (Created in BioRender: https://BioRender.com/rduvqd2). Guinea pigs (GPs, *N*=10/group) were either FTY720- or PBS-treated and then exposed two times to 15 ticks each. First-time exposed tick-naïve GPs were used as a tick attachment control. (**B, C**) Representative H&E skin cross-sections (>|5µm|<) of a

bioRxiv preprint doi: https://doi.org/10.1101/2025.08.22.671835; this version posted August 28, 2025. The copyright holder for this preprint (which was not certified by peer review) is the author/funder. This article is a US Government work. It is not subject to copyright under 17 USC 105 and is also made available for use under a CC0 license.

dermal biopsy at tick bite sites 48 h pta during the second tick exposure. 10x. (**B**) FTY720-treated GP showing mild epidermal hyperplasia and dermal edema but a minimal dermal infiltrate reminiscent of a first-time infested tick-naïve GP. (**C**) PBS-treated GP showing significant dermal inflammation and epidermal hyperplasia.

bioRxiv preprint doi: https://doi.org/10.1101/2025.08.22.671835; this version posted August 28, 2025. The copyright holder for this preprint (which was not certified by peer review) is the author/funder. This article is a US Government work. It is not subject to copyright under 17 USC 105 and is also made available for use under a CC0 license.

Figure S5

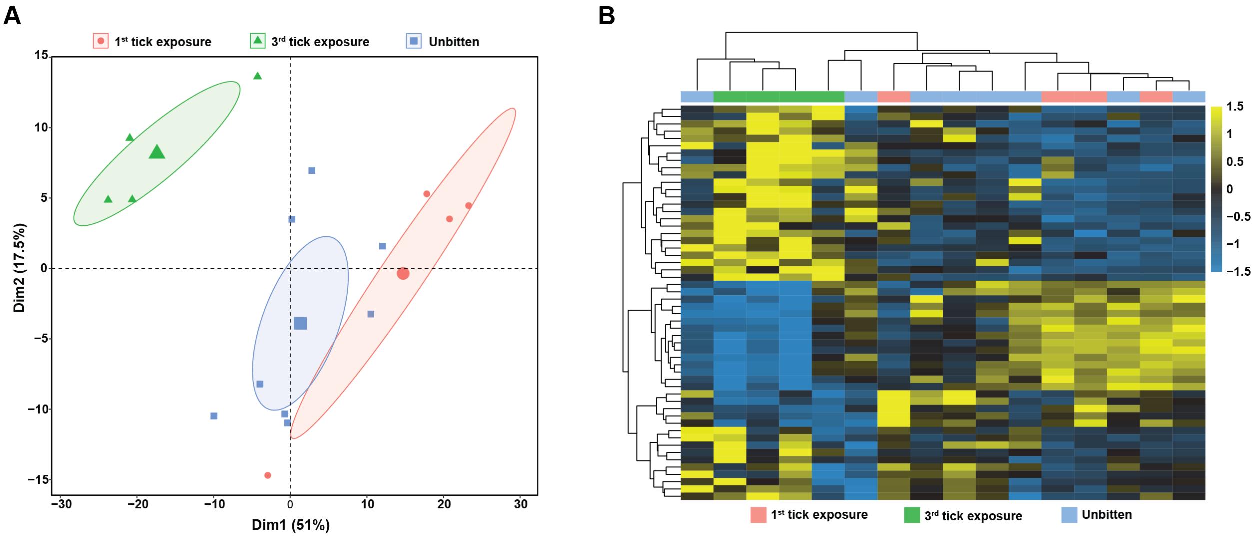

Figure S5. RNAseq analysis of 3 mm skin biopsies from tick bite sites during a first (*N*=4) and third (*N*=4) tick exposure, and unbitten skin (*N*=8). (**A**) PCA plot. (**B**) heatmap of the top 54 differentially expressed genes, either upregulated or downregulated, after applying a ±3 log2 fold change cutoff.

Figure S6

bioRxiv preprint doi: https://doi.org/10.1101/2025.08.22.671835; this version posted August 28, 2025. The copyright holder for this preprint (which was not certified by peer review) is the author/funder. This article is a US Government work. It is not subject to copyright under 17 USC 105 and is also made available for use under a CC0 license.

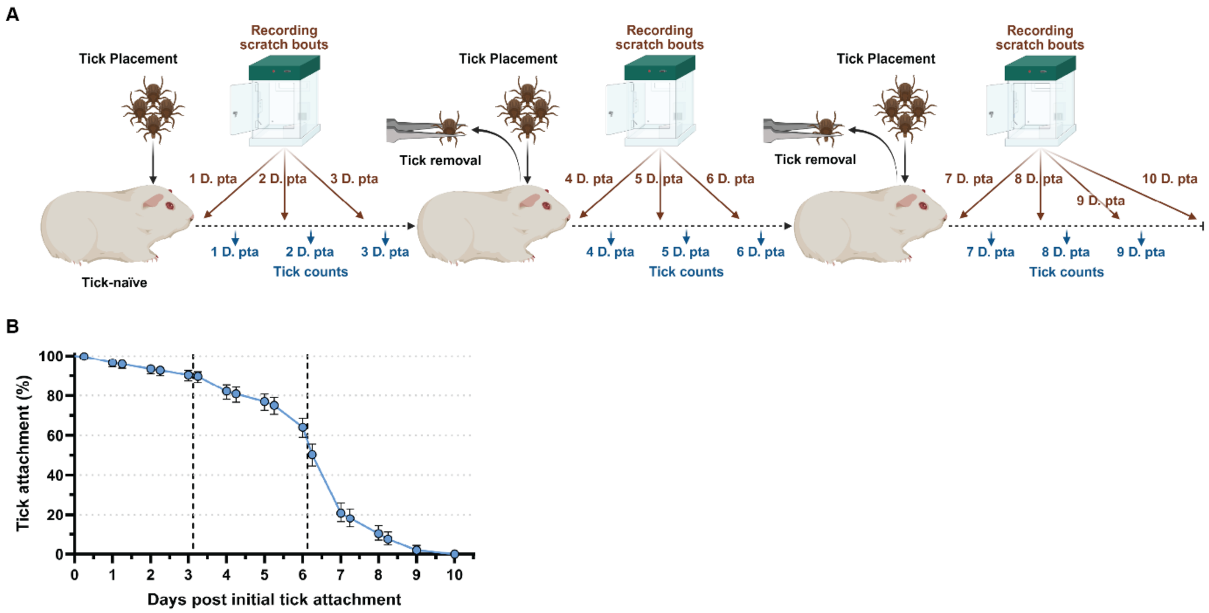

Figure S6. (**A**) Experimental design schematic of continuous tick exposure (Created in BioRender: https://BioRender.com/dtivqaj). (**B**) Smoothed Kaplan-Meyer plot showing probability of tick attachment (%) to continuously tick-exposed tick-naïve GPs up to 10 days post initial tick attachment (*N*=10 GPs). Dashed vertical lines indicate re-placement of ticks. 95% CI shown.

bioRxiv preprint doi: https://doi.org/10.1101/2025.08.22.671835; this version posted August 28, 2025. The copyright holder for this preprint (which was not certified by peer review) is the author/funder. This article is a US Government work. It is not subject to copyright under 17 USC 105 and is also made available for use under a CC0 license.

Figure S7

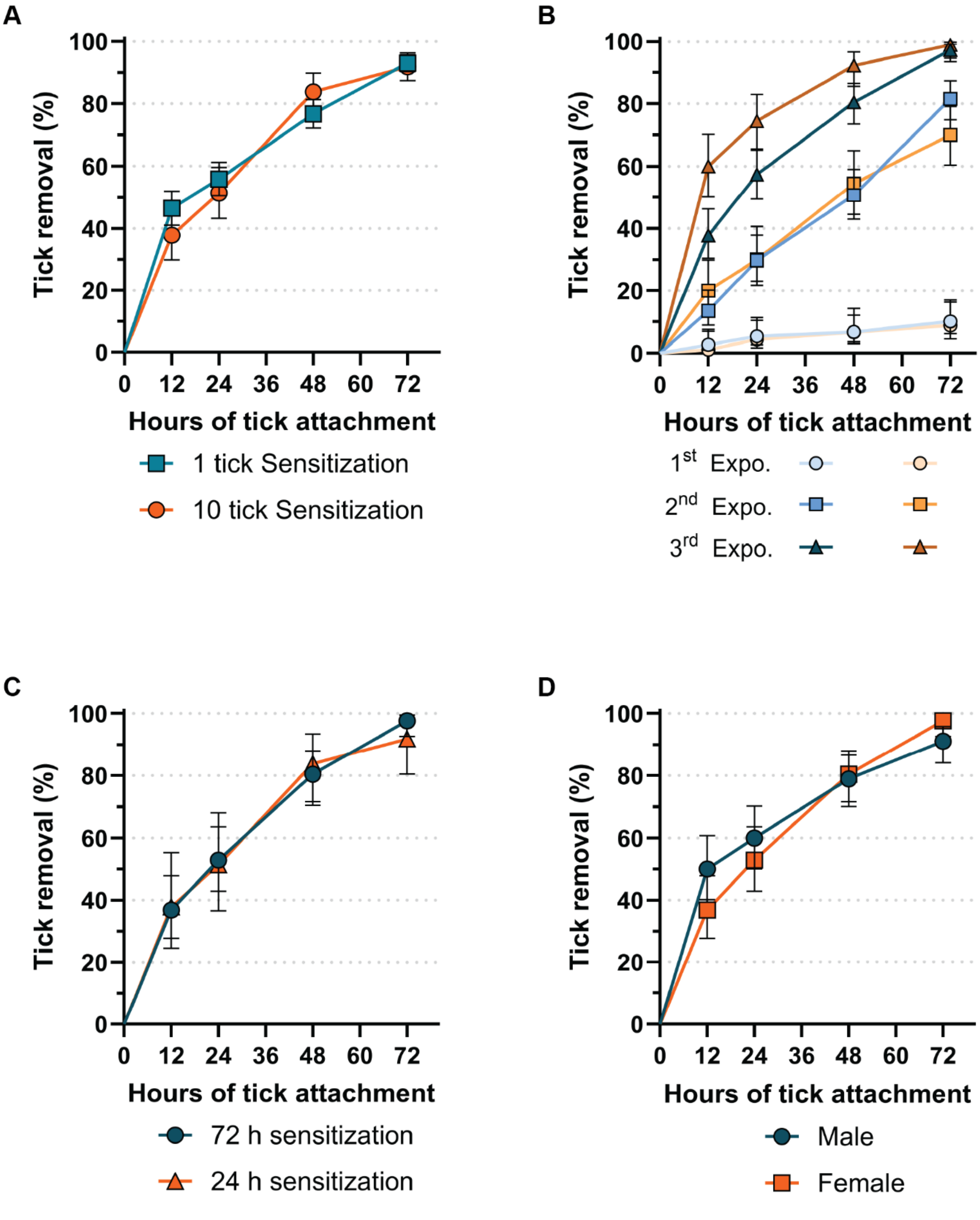

Figure S7. (**A-D**) Smoothed, inverted Kaplan-Meyer plot showing probability of tick removal (%). Analysis by Peto & Peto modification of the Gehan-Wilcoxon test. 95% CI shown. (**A**) 4th and 3rd weekly exposure of GPs sensitized with 1 and 10 ticks, respectively. p=0.782. (**B-D**) GPs

bioRxiv preprint doi: https://doi.org/10.1101/2025.08.22.671835; this version posted August 28, 2025. The copyright holder for this preprint (which was not certified by peer review) is the author/funder. This article is a US Government work. It is not subject to copyright under 17 USC 105 and is also made available for use under a CC0 license.

were exposed to 15 ticks per exposure. (**B**) Tick removal success in three times sensitized GPs exposed weekly (blue shades) or every three weeks (orange shades). 1st Exposure (Expo.): p=0.8787; 2nd Expo.: p=0.8787; 3rd Expo.: p=0.0006. (**C-D**) GPs were sensitized weekly. (**C**) Third exposure of GPs sensitized over 24 h or 72 h per exposure. p=0.8484. (**D**) Third exposure of male and female GPs. p=0.3898. For detailed statistics see supplementary report.

bioRxiv preprint doi: https://doi.org/10.1101/2025.08.22.671835; this version posted August 28, 2025. The copyright holder for this preprint (which was not certified by peer review) is the author/funder. This article is a US Government work. It is not subject to copyright under 17 USC 105 and is also made available for use under a CC0 license.

Figure S8

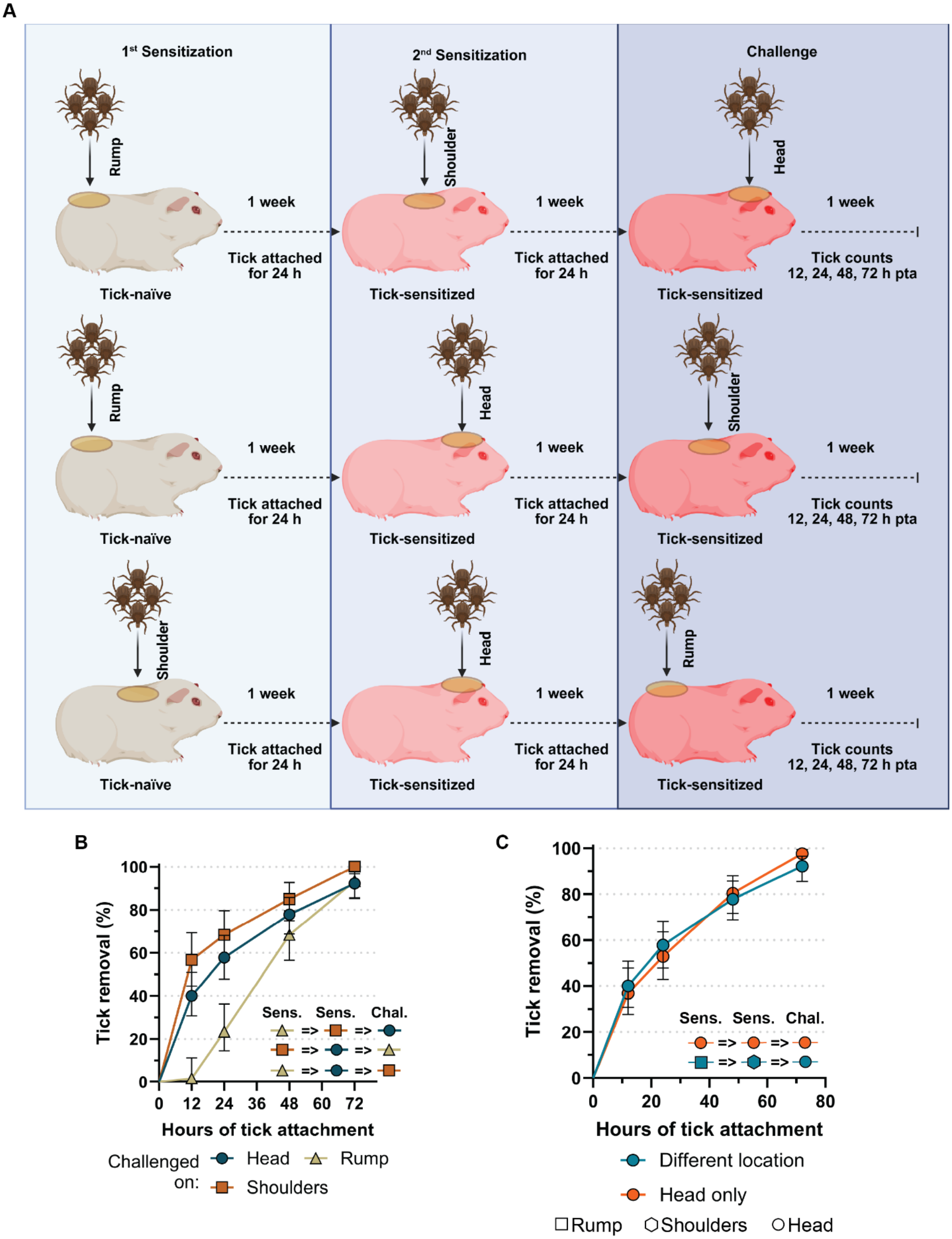

bioRxiv preprint doi: https://doi.org/10.1101/2025.08.22.671835; this version posted August 28, 2025. The copyright holder for this preprint (which was not certified by peer review) is the author/funder. This article is a US Government work. It is not subject to copyright under 17 USC 105 and is also made available for use under a CC0 license.

Figure S8. (**A**) Schematic of guinea pig (GP) sensitization and challenge depicting tick placement on different body locations (Created in BioRender: https://BioRender.com/y2smy29). (**B-C**) Smoothed, inverted Kaplan-Meyer plot showing probability of tick removal (%) in GPs sensitized weekly with 15 ticks. Analysis by Peto & Peto modification of the Gehan-Wilcoxon test. 95% CI is shown. (**B**) Shows results of tick challenge on different body location on GPs sensitized on other body locations than the challenge (see (**A**)). Head versus Rump: $p<0.0001$, Shoulders versus Rump: $p<0.0001$, Head versus Shoulders: p0.0483. (**C**) Tick removal success during a third exposure on the head in GP sensitized either on the same or different locations. $p=0.9403$. For more details see supplementary report.

bioRxiv preprint doi: https://doi.org/10.1101/2025.08.22.671835; this version posted August 28, 2025. The copyright holder for this preprint (which was not certified by peer review) is the author/funder. This article is a US Government work. It is not subject to copyright under 17 USC 105 and is also made available for use under a CC0 license.

Figure S9

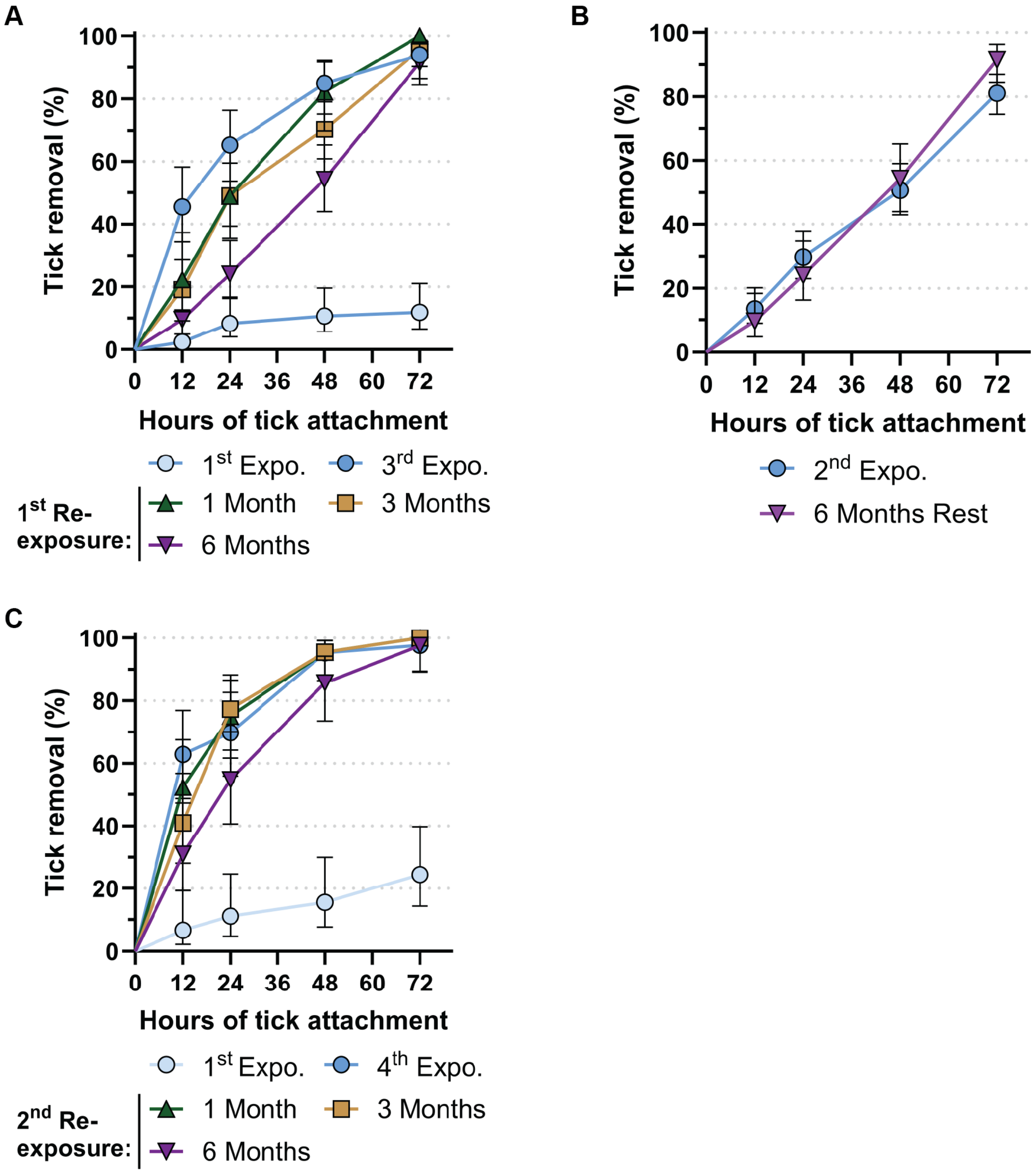

Fig. S9 (**A-C**) Smoothed, inverted Kaplan-Meyer plot showing probability of tick removal (%). Analysis by Peto & Peto modification of the Gehan-Wilcoxon test. 95% CI is shown. **(A-C)** Guinea pigs (GPs) were exposed to 15 ticks. (**A**) Tick removal success in GPs after 1, 3, and 6 month(s) of rest (no tick exposure) compared to a first exposure of tick-naïve GPs or a third exposure of weekly sensitized GPs. Third exposure (expo.) versus 1 Month [rest] (p=0.0536) /

bioRxiv preprint doi: https://doi.org/10.1101/2025.08.22.671835; this version posted August 28, 2025. The copyright holder for this preprint (which was not certified by peer review) is the author/funder. This article is a US Government work. It is not subject to copyright under 17 USC 105 and is also made available for use under a CC0 license.

versus 3 Months [rest] (p=0.0025) / versus 6 Months [rest] (p<0.0001). (**B**) Tick removal success of GPs during a second weekly exposure and after 6 months of no tick encounter. p=0.6281. (**C**) Tick removal success of the same GPs as in panel (**A**) during a subsequent tick-exposure a week later showing enhancement of tick removal success after long breaks from tick exposure. Forth exposure (expo.) versus 1 Month [rest] (p=0.6205) / versus 3 Months [rest] (p=0.3319) / versus 6 Months [rest] (p=0.03). For more details see supplementary report.

bioRxiv preprint doi: https://doi.org/10.1101/2025.08.22.671835; this version posted August 28, 2025. The copyright holder for this preprint (which was not certified by peer review) is the author/funder. This article is a US Government work. It is not subject to copyright under 17 USC 105 and is also made available for use under a CC0 license.

Table S1. Upregulated skin transcripts at the tick-bite site of tick-sensitized versus tick-tick-naive GP.

Transcript name and link to Uniprot Mouse or Rat ortholog	NCBI Identifier and link	Description	Log(2) fold change	P-value	Function	Reference
**Interleukins and other immune signaling products**						
Il19	100715337	interleukin 19	6.702564	5.25E-06	This cytokine is found to be preferentially expressed in monocytes. It can bind the IL20 receptor leading to the activation of transcription 3 (STAT3).	(*61*)
Fgf23	100717518	fibroblast growth factor 23	7.578738	0.000567	Involved in positive regulation of B cell proliferation	(*62*)
Osm	100724699	oncostatin M	4.402756	2.18E-09	Oncostatin M (OSM) contributes to extracellular matrix remodeling, hematopoiesis, differentiation, inflammatory response and proliferation.	(*63*)

bioRxiv preprint doi: https://doi.org/10.1101/2025.08.22.671835; this version posted August 28, 2025. The copyright holder for this preprint (which was not certified by peer review) is the author/funder. This article is a US Government work. It is not subject to copyright under 17 USC 105 and is also made available for use under a CC0 license.

Il13	100720118	interleukin 13	5.68252	0.009365	This gene encodes an immunoregulatory cytokine produced primarily by activated Th2 cells. This cytokine is involved in several stages of B-cell maturation and differentiation.	(*64*)
Cd28	100714966	CD28 molecule	3.558919	0.000983	Involved in immune response, T cell activation; T cell receptor signaling pathway; and in positive regulation of T cell proliferation;	(*65*)
Cd69	100727950	CD69 molecule	3.525725	5.66E-06	Expression Cd69 is induced upon activation of T lymphocytes and may play a role in their proliferation. Furthermore, the protein may act to transmit signals in natural killer cells and platelets.	(*66*)
Il24	100714873	interleukin 24	3.523712	0.002487	IL-24 induces rapid activation of Stat-1 and Stat-3 transcription factors.	(*67*)
Fcrlb	100715396	Fc receptor like B	3.512076	3.69E-05	FCRL1-5 could regulate different features of B-cell evolution such as development, differentiation, activation, antibody secretion and isotype switching.	(*68*)

bioRxiv preprint doi: https://doi.org/10.1101/2025.08.22.671835; this version posted August 28, 2025. The copyright holder for this preprint (which was not certified by peer review) is the author/funder. This article is a US Government work. It is not subject to copyright under 17 USC 105 and is also made available for use under a CC0 license.

Ms4a7	100719485	membrane spanning 4-domains A7	3.214635	0.000782	This family member is associated with mature cellular function in the monocytic lineage, and it may be a component of a receptor complex involved in signal transduction.	(*69*)
Cd38	106026535	ADP-ribosyl cyclase	3.388717	0.001823	Involved in positive regulation of B cell proliferation.	(*70, 71*)
Cd38	100726537	CD38 molecule	3.087273	2.44E-06	Involved in positive regulation of B cell proliferation	(*70, 71*)
G3V7I1	100735368	C-C motif chemokine 4	3.383439	0.000379	Involved in the immune response.	(*72*)
Q5Y4N7	100727811	adhesion G protein-coupled receptor E4P	4.339531	0.004216	Enables G protein-coupled receptor activity.	(*73*)
Fut7	100717385	fucosyltransferase 7	3.82816	0.000981	Involved in fucosylation.	(*74*)
**Cytotoxic T cells product**						
Clec1b	100726289	C-type lectin domain	3.592165	6.55E-09	Natural killer cells express multiple calcium-dependent lectin-like receptors that either inhibit or activate cytotoxicity and cytokine secretion	(*75*)

bioRxiv preprint doi: https://doi.org/10.1101/2025.08.22.671835; this version posted August 28, 2025. The copyright holder for this preprint (which was not certified by peer review) is the author/funder. This article is a US Government work. It is not subject to copyright under 17 USC 105 and is also made available for use under a CC0 license.

GRAB	100736180	granzyme B	6.598226	2.23E-06	Granzyme B (granzyme 2, cytotoxic T-lymphocyte-associated serine esterase 1)	(*76*)
GRAC	100719669	granzyme C	5.393386	0.000195	Granzyme C (granzyme 2, cytotoxic T-lymphocyte-associated serine esterase 1)	(*77*)
GRAB-Like2	100720219	granzyme B-like	4.93077	6.42E-07	Granzyme B (granzyme 2, -cytotoxic T-lymphocyte associated serine esterase 1)	(*76*)
**Chemotaxis**						
LOC100734903	100734903	C-C motif chemokine 3-like	3.424106	0.000368	Involved in eosinophil chemotaxis.	(*78*)
LOC100735187	100735187	C-C motif chemokine 3-like	3.132743	0.000621	Involved in eosinophil chemotaxis.	(*78*)
LOC100731901	100731901	alveolar macrophage chemotactic factor	3.996885	1.19E-08	Involved in immune response; involved in chemotaxis; and in defense response.	(*79*)
LOC100732171	100732171	platelet basic protein-like	5.654068	5.28E-05	Involved in chemotaxis	(*80*)
**Proteases**						
	Matrix metallopeptidases					

bioRxiv preprint doi: https://doi.org/10.1101/2025.08.22.671835; this version posted August 28, 2025. The copyright holder for this preprint (which was not certified by peer review) is the author/funder. This article is a US Government work. It is not subject to copyright under 17 USC 105 and is also made available for use under a CC0 license.

Mmp3	100729101	stromelysin-1	3.872797	1.80E-11	Involved in collagen catabolic process; and in extracellular matrix organization.	(*81*)
Mmp7	Mmp4	matrix metallopeptidase 7	4.247669	4.95E-07	Involved in collagen catabolic process; and in extracellular matrix organization.	(*82*)
Mmp8	100724670	matrix metallopeptidase 8, neutrophil collagenase	3.120398	2.30E-05	Involved in collagen catabolic process; and in extracellular matrix organization.	(*83*)
Mmp10	100727690	matrix metallopeptidase 10	4.925036	1.64E-10	Involved in collagen catabolic process; and in extracellular matrix organization.	(*84*)
Mmp13	100731756	matrix metallopeptidase 13	3.183712	1.77E-05	Involved in collagen catabolic process; involved in extracellular matrix organization.	(*85*)
Other peptidases						
Kelz	100729473	Kell metallo-endopeptidase	4.564278	4.43E-05	Hydrolyses bradykinin and neuropeptides.	(*86, 87*)

Prss29	100724332	Serine protease 29-like	6.958842	1.79E-05	Involved in proteolysis.	(*88*)
**Prostanoid catabolism**						

bioRxiv preprint doi: https://doi.org/10.1101/2025.08.22.671835; this version posted August 28, 2025. The copyright holder for this preprint (which was not certified by peer review) is the author/funder. This article is a US Government work. It is not subject to copyright under 17 USC 105 and is also made available for use under a CC0 license.

CP4FE	100733735	cytochrome P450 4F3-like	5.74894	7.25E-05	Cytochrome P450 4F3 (CYP4F3), originally identified as one of the leukotriene B4 ω-hydroxylases, participate in the metabolism of various endobiotics, as well as some xenobiotics.	(*89*)
**Innate immunity**						
APOBEC-1	100730186	APOBEC-1-like	5.978293	0.007912	Nuclear C to U RNA editing - many of these candidate RNAs are involved in cytokine signaling; anti-viral.	(*90*)
Oasl	100734390	2'-5'-oligoadenylate synthetase like	3.94653	4.14E-05	Involved in defense response to virus; involved in negative regulation of viral genome replication; involved in regulation of ribonuclease activity	(*91*)
NGAL	100722819	neutrophil gelatinase-associated lipocalin-like	6.307814	0.0051	Enables iron ion binding activity. Involved in innate immune response, positive regulation of cold-induced thermogenesis; and siderophore transport.	(*92*)
Kunitz serine protease inhibitor						

bioRxiv preprint doi: https://doi.org/10.1101/2025.08.22.671835; this version posted August 28, 2025. The copyright holder for this preprint (which was not certified by peer review) is the author/funder. This article is a US Government work. It is not subject to copyright under 17 USC 105 and is also made available for use under a CC0 license.

Spint3	101787904	Protease inhibitors-like, Kunitz type, 3	3.861328	3.76E-05	Protease inhibition.	(*93*)
